# Navigating the Therapeutic Landscape: A Comprehensive Review of Platelet-Rich Plasma and Bone Marrow Aspirate Concentrate in Knee Osteoarthritis

**DOI:** 10.7759/cureus.54747

**Published:** 2024-02-23

**Authors:** Prashanth Balusani, Sandeep Shrivastava, Aditya Pundkar, Prathamesh Kale

**Affiliations:** 1 Orthopaedics and Traumatology, Jawaharlal Nehru Medical College, Datta Meghe Institute of Medical Science, Wardha, IND; 2 Orthopaedic Surgery, Jawaharlal Nehru Medical College, Datta Meghe Institute of Medical Science, Wardha, IND; 3 Orthopaedics, Jawaharlal Nehru Medical College, Datta Meghe Institute of Medical Science, Wardha, IND

**Keywords:** safety considerations, clinical efficacy, regenerative medicine, knee osteoarthritis, bone marrow aspirate concentrate (bmac), platelet-rich plasma (prp)

## Abstract

This comprehensive review provides an in-depth analysis of platelet-rich plasma (PRP) and bone marrow aspirate concentrate (BMAC) as potential treatments for knee osteoarthritis. It explores their mechanisms of action, clinical efficacy, safety considerations, and the importance of personalised treatment approaches. The review highlights promising findings regarding the ability of PRP and BMAC to alleviate symptoms, improve joint function, and potentially slow disease progression. It emphasises the need for further research into long-term outcomes, direct comparative studies, protocol standardisation, biomarker identification, and cost-effectiveness assessments to enhance clinical practice. While the review does not directly compare PRP and BMAC, it provides valuable insights into their respective roles in knee osteoarthritis management. The review aims to contribute to evidence-based advancements in regenerative therapies for knee osteoarthritis by addressing critical research priorities and refining treatment strategies.

## Introduction and background

Osteoarthritis is a formidable challenge in musculoskeletal disorders, particularly affecting the knee joint. The insidious progression of knee osteoarthritis, characterised by the degeneration of articular cartilage, subchondral bone changes, and inflammation, substantially burdens individuals' quality of life worldwide. With an ageing population and an increasing prevalence of obesity, the incidence of knee osteoarthritis is on the rise, underscoring the pressing need for innovative therapeutic strategies [1]. Understanding the intricacies of knee osteoarthritis necessitates a grasp of its multifaceted aetiology. Osteoarthritis is not merely a consequence of wear and tear but involves a complex interplay of genetic, biomechanical, and inflammatory factors. The progressive degradation of cartilage, alterations in subchondral bone density, and the activation of synovial inflammation collectively contribute to the pathogenesis of knee osteoarthritis [2].

Knee osteoarthritis significantly impacts individuals' daily activities, leading to pain, stiffness, and reduced mobility. Beyond the personal toll, it exerts a substantial socioeconomic burden through increased healthcare costs and productivity losses. As the population ages and lifestyles evolve, the prevalence of knee osteoarthritis continues to escalate, making it imperative to explore novel and effective therapeutic avenues [3].

The landscape of knee osteoarthritis management is evolving, shifting from traditional symptomatic relief to disease-modifying interventions. In this context, emerging therapeutic approaches offer promise for altering the course of the disease rather than merely mitigating symptoms. Among these, platelet-rich plasma (PRP) and bone marrow aspirate concentrate (BMAC) have garnered significant attention for their potential to promote tissue regeneration and modulate the inflammatory milieu [4].

This comprehensive review aims to navigate the therapeutic landscape of knee osteoarthritis, with a specific focus on PRP and BMAC. By synthesising current knowledge, clinical evidence, and emerging trends, the review seeks to provide a holistic understanding of the efficacy, mechanisms of action, and safety considerations associated with these regenerative therapies. Furthermore, the review will explore the comparative aspects of PRP and BMAC, shedding light on their respective roles in reshaping the paradigm of knee osteoarthritis management. Through this exploration, the review aspires to contribute valuable insights that guide clinical decision-making, inspire further research, and ultimately enhance the therapeutic options available for individuals grappling with knee osteoarthritis.

## Review

PRP in knee osteoarthritis

PRP has emerged as a promising and innovative therapeutic approach in regenerative knee osteoarthritis medicine. This treatment modality involves the extraction and concentration of platelets from the patient's blood, creating a potent elixir rich in growth factors and bioactive proteins integral to tissue repair and regeneration [5]. These elements, including platelet-derived growth factor (PDGF), transforming growth factor-beta (TGF-β), and others, play pivotal roles in orchestrating cellular processes essential for healing damaged tissues. The application of PRP in knee osteoarthritis is rooted in the objective of harnessing the body's innate healing mechanisms. By delivering a concentrated source of platelets and associated bioactive molecules directly to the site of injury or degeneration within the knee joint, PRP aims to stimulate and accelerate the natural regenerative processes. This therapeutic approach offers a minimally invasive alternative to traditional treatments for knee osteoarthritis, such as pain medications or invasive surgical procedures. It underscores a paradigm shift towards utilising the body's resources to promote healing and alleviate symptoms [5]. Understanding PRP's fundamental principles and applications is paramount for evaluating its efficacy and potential in managing knee osteoarthritis [6]. This entails a comprehensive grasp of the underlying biology and molecular mechanisms through which PRP exerts its therapeutic effects. Clinicians and researchers must explore the components of PRP and the dynamic processes it triggers within the joint. This knowledge is essential for informed decision-making, allowing healthcare professionals to assess the appropriateness of PRP for specific patients, tailor treatment protocols, and contribute to the ongoing evolution of regenerative medicine strategies for knee osteoarthritis. As the field advances, a deeper understanding of PRP's applications will likely pave the way for increasingly targeted and effective interventions in managing knee osteoarthritis.

Mechanisms of Action

PRP's therapeutic efficacy in knee osteoarthritis is intricately linked to its complex composition, prominently featuring an array of growth factors. Among these, PDGF, TGF-β, and insulin-like growth factor (IGF) emerge as vital bioactive substances with pivotal roles in tissue repair and regeneration. PDGF plays a crucial role in cell proliferation and angiogenesis, while TGF-β is renowned for its involvement in cell differentiation and the synthesis of extracellular matrix components. Conversely, IGF exerts potent effects on cellular metabolism and tissue development [7]. The synergistic action of these growth factors is fundamental to the therapeutic mechanisms of PRP. Collectively, they stimulate and orchestrate intricate cellular processes, promoting the repair of damaged tissues within the knee joint. Central to PRP's therapeutic impact is its capacity to reduce inflammation, a hallmark feature of osteoarthritis. By modulating the immune response, PRP mitigates the inflammatory cascade associated with the disease, thereby alleviating pain and potentially impeding the progression of joint degeneration [8]. Beyond its role in promoting tissue repair and mitigating inflammation, PRP demonstrates notable anti-catabolic properties. Osteoarthritis disrupts the delicate balance of joint homeostasis, leading to articular cartilage degradation. Through its ability to modulate catabolic processes, PRP contributes to restoring this balance. It acts as a regulatory force, counteracting the destructive pathways associated with osteoarthritis and fostering an environment conducive to cartilage regeneration [9]. Understanding the intricate mechanisms by which PRP influences joint homeostasis is paramount to unlocking its full therapeutic potential. This understanding underscores the rationale for its application in knee osteoarthritis and guides the refinement of treatment protocols. As research delves deeper into PRP's molecular and cellular intricacies, it paves the way for more targeted and personalised approaches, positioning PRP as a promising avenue in the quest for effective regenerative therapies for knee osteoarthritis [10].

Clinical Studies and Efficacy

PRP has been considered a potential treatment for knee osteoarthritis due to its potential to reduce pain and improve function [11]. However, the clinical efficacy of PRP in knee osteoarthritis treatment remains controversial, with experts suggesting more research is needed [11]. A study involving 153 patients found that PRP injections provided more significant benefits than hyaluronic acid in terms of long-term discomfort [12]. Another study with a follow-up period of six to eight weeks reported improvements in pain, stiffness, and function [11]. A study published in The Journal of International Medical Research examined five clinical trials, including 320 patients, and found that intra-articular PRP injection is an effective knee osteoarthritis treatment that can reduce post-operative pain, improve locomotor function, and increase patient satisfaction [13]. A study involving 517 consecutive patients found that the effectiveness of PRP therapy for knee osteoarthritis treatment was approximately 60% and depended on the severity of the condition [13]. A double-blinded, placebo-controlled, randomised clinical trial published in BMC Musculoskeletal Disorders found conflicting efficacy for knee osteoarthritis treatment [14]. PRP injections show potential for treating knee osteoarthritis, but more research is needed to confirm their effectiveness and safety. While some studies have reported positive outcomes, others have expressed concerns about standardising PRP preparations and techniques. Current guidelines do not recommend PRP for knee osteoarthritis treatment, but it could become a helpful treatment option with further research [12,15].

Variations in PRP Preparations

Variations in PRP preparations constitute a pivotal aspect of its clinical utility in managing knee osteoarthritis. The diverse landscape of PRP formulations, characterised by differences in platelet concentration, white blood cell content, and activation methods, introduces a nuanced dimension to their therapeutic application. The significance of exploring these variations lies in their potential to profoundly influence the therapeutic properties of PRP and, consequently, treatment outcomes [5]. Platelet concentration within PRP preparations is a critical parameter that impacts the abundance of growth factors and other bioactive substances essential for tissue regeneration. The inclusion or exclusion of white blood cells further adds complexity to PRP formulations, as these cells can modulate the immune response and contribute to regenerative processes. Additionally, the choice of activation methods, such as thrombin or calcium chloride, can influence the release of growth factors from platelets, affecting the overall efficacy of PRP [16]. Recognising the impact of these variations is crucial for tailoring PRP treatment protocols to individual patient needs. Patient-specific factors, including the stage of osteoarthritis and the overall health profile, can influence the optimal PRP formulation. For instance, specific formulations with higher platelet concentrations might be more suitable for advanced stages of osteoarthritis, where robust tissue regeneration is paramount. Conversely, formulations with lower platelet concentrations and reduced white blood cell content may be preferred for patients with milder symptoms or specific sensitivities [17]. Understanding the nuances of PRP preparations empowers clinicians to adopt a personalised approach, optimising the therapeutic benefits for each patient. By aligning PRP formulations with the unique requirements of individual cases, clinicians can enhance treatment efficacy and potentially contribute to more favourable outcomes. As research unravels the intricate details of PRP composition and its impact on knee osteoarthritis, the customisation of PRP preparations holds promise for advancing precision medicine within regenerative therapies [18].

Safety Considerations

While PRP is generally recognised as a safe therapeutic option, a meticulous analysis of safety considerations is paramount. This comprehensive examination involves a thorough investigation into potential adverse events that may arise following PRP administration, including but not limited to infection, bleeding, or allergic reactions. These safety considerations are critical in evaluating PRP's suitability as a therapeutic intervention for knee osteoarthritis [19]. Understanding the long-term safety profile of PRP in the context of knee osteoarthritis is an essential dimension of this analysis. While short-term safety assessments provide valuable insights, a more extended and nuanced perspective is required to establish PRP as a sustainable and reliable therapeutic option. Long-term safety considerations are crucial for evaluating the durability of PRP's effects and monitoring for any delayed adverse events that may arise over an extended period [19]. The assessment of PRP safety encompasses the inherent risks associated with the procedure itself and an exploration of patient-specific factors that may influence the likelihood of complications. Individual variations, such as pre-existing medical conditions, medication regimens, or unique physiological characteristics, can play a role in shaping the safety profile of PRP for a particular patient. Therefore, a personalised risk assessment is warranted to ensure that the benefits of PRP therapy outweigh the potential risks for each individual [20]. A comprehensive understanding of the safety profile is vital for PRP's responsible and ethical application in the clinical setting. This involves informing patients of potential risks and implementing rigorous protocols and standards to mitigate these risks. Continuous monitoring and reporting of adverse events contribute to ongoing improvements in safety protocols, fostering a culture of accountability and diligence in using PRP for knee osteoarthritis. Ultimately, this commitment to safety ensures that PRP remains a trustworthy and ethically administered therapeutic option in regenerative medicine [19].

BMAC in knee osteoarthritis

BMAC has emerged as a notable and increasingly studied regenerative therapy for knee osteoarthritis. What sets BMAC apart from other treatments is its unique strategy of harnessing the inherent regenerative potential residing in the patient's bone marrow, primarily through mesenchymal stem cells (MSCs) and growth factors [21]. This innovative approach represents a departure from traditional treatments, seeking to directly address the degenerative processes associated with osteoarthritis by delivering a concentrated mixture of regenerative cells and signaling molecules directly to the affected joint. BMAC's utilisation of MSCs is particularly noteworthy. These stem cells can differentiate into various cell types, including chondrocytes responsible for cartilage formation. By incorporating MSCs into the treatment, BMAC aims to alleviate symptoms and actively contribute to regenerating damaged joint tissues. Additionally, including growth factors further enhances BMAC's regenerative potential, as these bioactive molecules play crucial roles in tissue repair and modulating the local environment within the joint [22].

The novel approach of BMAC aligns with a broader shift towards regenerative medicine strategies that leverage the body's resources for healing. By directly tapping into the regenerative potential in the patient's bone marrow, BMAC presents a promising avenue for addressing the root causes of knee osteoarthritis. Understanding the fundamental principles and applications of BMAC is imperative for assessing its efficacy and safety in managing knee osteoarthritis [22]. This entails comprehensively comprehending the biological mechanisms through which BMAC exerts its therapeutic effects and evaluating its potential benefits and risks. As research into BMAC continues to evolve, the knowledge gained will contribute to refining treatment protocols, optimising patient selection criteria, and enhancing the overall effectiveness of regenerative therapies for knee osteoarthritis. The ongoing exploration of BMAC's applications holds the potential to reshape the landscape of osteoarthritis management, offering a promising avenue for those seeking alternatives to traditional treatments [22].

Cellular Components and Functions

The regenerative potential of BMAC hinges significantly on its cellular components, with a central role played by MSCs. These versatile cells are integral to BMAC's therapeutic efficacy due to their unique capability to differentiate into diverse cell types, most notably chondrocytes, the primary cells responsible for the formation and maintenance of cartilage tissue. Including MSCs in BMAC infusions holds immense promise for promoting the regeneration of damaged or degenerated joint tissues [23]. Beyond MSCs, BMAC also encompasses a rich milieu of growth factors and cytokines. These bioactive molecules contribute to the orchestration of tissue repair processes and play a vital role in modulating the inflammatory environment within the joint. The interplay between these cellular components creates a dynamic environment conducive to healing and regeneration, addressing critical aspects of the pathological changes associated with knee osteoarthritis [24].

By delving into the specific functions of these cellular constituents, a more profound understanding emerges regarding the mechanisms through which BMAC exerts its therapeutic effects. MSCs, with their capacity for differentiation, offer the potential to replace or repair damaged cartilage, thereby addressing one of the fundamental challenges in osteoarthritis. Simultaneously, the growth factors and cytokines present in BMAC contribute to a multifaceted approach, promoting tissue repair and modulating the inflammatory milieu within the joint [25]. Understanding the intricate interplay between MSCs and growth factors is pivotal for deciphering the nuanced mechanisms by which BMAC influences joint regeneration. This knowledge is foundational for clinicians and researchers seeking to optimise BMAC-based treatments for knee osteoarthritis. It provides a roadmap for tailoring therapeutic strategies, optimising treatment protocols, and advancing the field of regenerative medicine towards more targeted and effective interventions for joint health. As research in this area continues to unfold, a deeper understanding of these cellular mechanisms can revolutionise the landscape of osteoarthritis management [26].

Clinical Studies and Efficacy

BMAC has emerged as a promising therapeutic option for addressing symptomatic knee osteoarthritis, primarily due to its abundant supply of MSCs and growth factors [21,27]. The therapeutic potential of BMAC stems from the unique properties of MSCs, which exhibit both anti-inflammatory and regenerative capabilities, offering a multifaceted approach to addressing the underlying biochemical pathology of arthritis [27]. A systematic review involving 299 knees with an average follow-up of 12.9 months revealed compelling outcomes. Of the 36 patient-reported outcomes analysed, 34 (94.4%) demonstrated significant improvement from baseline to the latest follow-up, indicating the positive impact of BMAC on knee osteoarthritis symptoms [28]. This suggests a noteworthy potential for BMAC to influence patient-reported outcomes positively over the short- to medium-term.

Furthermore, a study published in BMC Musculoskeletal Disorders affirmed the safety and efficacy of intra-articular BMAC injections in treating pain and enhancing functionality in patients with symptomatic knee osteoarthritis [29]. This further supports the notion that BMAC can be a well-tolerated and effective intervention for managing the symptoms associated with knee osteoarthritis. In a prospective comparative clinical trial comparing BMAC with adipose-derived stem cells (ADSCs), both BMAC and ADSC intra-articular injections significantly improved pain and functional outcomes at a six-month follow-up in knee osteoarthritis patients [30]. This comparative analysis underscores the potential efficacy of BMAC and positions it alongside other regenerative therapies in the treatment landscape for knee osteoarthritis.

While these studies suggest promising outcomes, it is emphasised that more research is necessary to confirm the effectiveness and safety of BMAC in treating knee osteoarthritis. The call for well-designed, randomised, controlled clinical trials is essential to elucidate the exact mechanisms of BMAC and to establish its potential benefits with a high degree of certainty in the context of knee osteoarthritis treatment [21]. Despite the positive findings reported in various studies, a rigorous scientific approach is crucial to provide conclusive evidence and ensure that BMAC can be integrated into clinical practice as a safe and effective therapeutic option for knee osteoarthritis.

Variations in BMAC Processing

The processing of BMAC is a multifaceted procedure encompassing several critical steps, each with potential implications for the final product's cellular and growth factor composition. These key processing stages typically involve aspiration of bone marrow, concentration of the aspirate to isolate desired components, and activation to prepare the concentrate for therapeutic application. The intricacies involved in these steps are pivotal in determining the quality and effectiveness of the BMAC product [31]. Variations in the aspiration process can impact the quantity and types of cells retrieved from the bone marrow, including MSCs that are central to BMAC's regenerative potential. The subsequent concentration step isolates and enriches specific cellular components, such as MSCs and platelets, contributing to tissue repair. The activation process is also crucial for preparing the concentrated material for therapeutic use, influencing the release of growth factors and cytokines [32].

Understanding the nuances of BMAC processing is paramount for optimising therapeutic protocols. The variability introduced at each processing step can directly affect the concentration and potency of the cellular and growth factor components within the final BMAC product. This nuanced comprehension empowers clinicians to tailor BMAC treatments based on individual patient characteristics and specific attributes of knee osteoarthritis [33]. By customising processing protocols, clinicians can optimise the regenerative potential of BMAC, tailoring treatments to each patient's unique needs. Factors such as disease severity, patient age, and the specific joint structures affected can be considered in the processing strategy, maximising the therapeutic impact of BMAC. This level of personalisation in treatment approaches contributes to the ongoing refinement of regenerative therapies for knee osteoarthritis, aligning interventions more closely with the diverse and dynamic nature of each patient's condition [34].

Safety Considerations

While BMAC is generally considered a safe therapeutic option, a thorough and nuanced analysis of safety considerations is paramount. This comprehensive examination encompasses scrutiny of potential adverse events that may arise following BMAC administration, including but not limited to infection, bleeding, or unintended tissue formation. Additionally, evaluating the long-term safety profile of BMAC in knee osteoarthritis is crucial for establishing its role as a reliable and sustainable therapeutic option [35]. The assessment of BMAC safety involves a multifaceted approach. Firstly, understanding the inherent risks associated with the procedure itself is essential. This includes potential complications related to the extraction of bone marrow, the concentration process, and the subsequent injection of the concentrate into the affected joint. Rigorous adherence to standardised protocols and stringent aseptic techniques during these procedural steps is crucial to minimising the risk of adverse events [35].

Secondly, consideration of patient-specific factors is paramount. Individual variations, such as pre-existing medical conditions, medication regimens, and unique physiological characteristics, may influence the likelihood of complications. A personalised risk assessment, considering the patient's overall health status, is warranted to ensure that the benefits of BMAC therapy outweigh the potential risks for each individual. Evaluating the long-term safety profile of BMAC is particularly important as it provides insights into the durability of treatment effects and the potential for delayed adverse events. Monitoring patients over an extended period allows for identifying any latent safety concerns. It contributes to establishing the reliability and sustainability of BMAC as a therapeutic option for knee osteoarthritis [35]. A comprehensive understanding of the safety profile is not only crucial for the well-being of patients but also ensures the responsible and ethical application of BMAC in the clinical setting. Continuous vigilance, reporting of adverse events, and adherence to best practices foster a culture of safety and accountability when using BMAC for knee osteoarthritis [36].

Comparative analysis of PRP and BMAC

Efficacy Comparison

More comprehensive studies are needed to compare the outcomes of PRP and BMAC in the treatment of knee osteoarthritis. A study comparing PRP and BMAC outcomes in bone and cartilage found no significant difference between the two groups and empty lyophilised bone controls, as all patients reported relieved knee pain and a full range of motion [37]. The biological differences between BMAC and PRP, including the concentration of leukocytes, have been examined. Still, current protocols have yet to reach consensus regarding the methodology used, which could weaken the data quality and pose a challenge for comparative analyses [29]. Neither the American College of Rheumatology nor the Arthritis Foundation recommends PRP treatment for knee and hip osteoarthritis, citing concerns about standardising preparations and techniques [12]. A well-analyzed study found that BMAC showed more PDGF, TGF-β, and vascular endothelial growth factor (VEGF) in comparison to PRP preparation with no statistically significant difference [38]. While some individual studies have compared PRP and BMAC in treating knee osteoarthritis, there needs to be more consensus and comprehensive evidence to determine the superiority of one treatment over the other. More well-designed, randomised clinical trials with well-defined controls and parameters are needed to consolidate these treatments' efficacy, both individually and in combination [12,29,37,38].

Safety Comparison

The safety comparison between PRP and BMAC in the treatment of knee osteoarthritis is an area of ongoing research, and the available evidence is limited. Clinical data has shown that both PRP and BMAC are safe and have shown positive results for cartilage and bone injuries [37]. PRP is considered an experimental treatment for managing pain related to knee osteoarthritis, and while early trials have shown promising results, current guidelines do not recommend its widespread use [15]. A retrospective comparative study found that intra-articular autologous BMAC therapy is safe and provides more relief to patients with symptomatic knee osteoarthritis compared to PRP [29]. A well-analysed study found that BMAC showed more PDGF, TGF-β, and VEGF in comparison to PRP preparation with no statistically significant difference [38]. While both PRP and BMAC are safe for treating knee osteoarthritis, the available evidence is limited, and more research is needed to make a comprehensive safety comparison between the two treatments [15,29,37,38].

Patient Selection Criteria

The patient selection criteria for PRP and BMAC in the treatment of knee osteoarthritis are crucial for achieving optimal outcomes. However, the existing literature highlights the need for more comprehensive studies and consensus on the methodology for the preparation, dosage, and administration of PRP and BMAC, which could weaken the quality of data and pose a challenge for comparative analyses [29]. While some studies have suggested that BMAC may be more beneficial than PRP, especially in milder osteoarthritis grades, it is essential to consider the limitations of the current evidence, including the small sample sizes and the lack of consensus on the treatment protocols [29]. Additionally, the safety and efficacy of both PRP and BMAC have been demonstrated in treating knee osteoarthritis, but further research is needed to establish clear patient selection criteria and comparative effectiveness [39]. The available literature emphasises the need for well-designed studies with larger sample sizes and standardised protocols to determine the optimal patient selection criteria for PRP and BMAC in treating knee osteoarthritis. Until then, individual patient characteristics, disease severity, and treatment goals should be carefully considered when selecting the most appropriate treatment option.

Cost-Effectiveness Considerations

The cost-effectiveness considerations of PRP and BMAC in the treatment of knee osteoarthritis need to be well-established in the literature. More studies are needed to compare the cost-effectiveness of PRP and BMAC, and the available evidence is limited to clinical outcomes and safety profiles [29,37,39,40]. While some studies have suggested that BMAC may be more beneficial than PRP, especially in milder osteoarthritis grades, it is essential to consider the limitations of the current evidence, including the small sample sizes and the lack of consensus on the treatment protocols [29]. Additionally, the safety and efficacy of both PRP and BMAC have been demonstrated in treating knee osteoarthritis, but further research is needed to establish clear patient selection criteria and comparative effectiveness [39,40]. The available literature must provide sufficient evidence to make a comprehensive cost-effectiveness comparison between PRP and BMAC in treating knee osteoarthritis. Further research is needed to determine the optimal treatment protocols and evaluate these treatments' cost-effectiveness.

Combination of therapies and emerging approaches

Combining PRP and BMAC

The combination of PRP and BMAC is a synergistic and comprehensive approach to addressing knee osteoarthritis. This innovative strategy leverages the distinct regenerative properties of PRP and BMAC, aiming to capitalise on the interplay between PRP's rich array of growth factors and BMAC's potent MSCs. This collaborative action introduces a multi-faceted regenerative potential that holds promise for enhancing tissue repair and mitigating the progression of knee osteoarthritis [40]. The biological synergy between PRP and BMAC is particularly noteworthy. PRP, with its abundance of growth factors like PDGF, TGF-β, and IGF, provides a rich signalling environment for cell activation and tissue regeneration. When combined with BMAC, which contains MSCs capable of differentiating into various cell types, including chondrocytes, the collaboration creates a dynamic and versatile platform for joint healing [41].

Elucidating the intricate biological mechanisms that underlie this combination is pivotal for clinicians seeking to optimise treatment protocols. Understanding how the growth factors from PRP synergise with the regenerative potential of MSCs from BMAC allows for a targeted and personalised approach. By tailoring treatment protocols based on these biological insights, clinicians can harness the maximum therapeutic benefit for patients with knee osteoarthritis [42]. This synergistic approach could address the symptoms and underlying causes of knee osteoarthritis. It offers a comprehensive strategy for promoting tissue repair, modulating the inflammatory environment, and potentially altering the disease trajectory. As research continues to unveil the intricacies of this combination therapy, it lays the groundwork for advancements in regenerative medicine, shaping the future of holistic and personalised interventions for knee osteoarthritis.

Other Emerging Therapies in Knee Osteoarthritis

Gene therapy: Gene therapy represents a cutting-edge approach in regenerative medicine for joint tissues. This involves introducing specific genetic material into cells to modify or enhance their regenerative capacity. In the context of joint tissues, gene therapy may target cells involved in cartilage or synovial tissue repair. For example, researchers are exploring the delivery of genes encoding growth factors or proteins that stimulate chondrogenesis, the process of forming new cartilage. By manipulating the genetic expression of cells within the joint, gene therapy can potentially promote more effective and sustained tissue regeneration, offering a novel avenue for addressing the underlying causes of joint degeneration in conditions like osteoarthritis [43].

Exosome therapy: Exosome therapy uses exosomes, small vesicles derived from stem cells, to deliver bioactive molecules and promote tissue regeneration within the joint. Stem-cell-derived exosomes are enriched with growth factors, microRNAs, and other signalling molecules that play crucial roles in cell communication and tissue repair. These exosomes can be harnessed to modulate cellular processes in the joint, promoting anti-inflammatory effects, inhibiting apoptosis, and enhancing the regenerative potential of resident cells. This approach offers a more targeted and potentially safer alternative to traditional cell-based therapies, as exosomes can exert therapeutic effects without requiring direct cell transplantation [44].

Biological scaffolds: Biological scaffolds involve the application of supportive structures that facilitate tissue repair and provide structural support within the joint. These scaffolds can be derived from natural sources, such as extracellular matrix components or synthetic materials designed to mimic the properties of native tissue. In joint regeneration, biological scaffolds can be engineered to create a conducive environment for cell attachment, proliferation, and differentiation. These scaffolds may serve as a three-dimensional framework for cells to populate and organise, promoting the regeneration of damaged or degenerated joint tissues. Biological scaffolds are particularly relevant for addressing structural defects in cartilage or meniscus, offering a promising strategy for enhancing the outcomes of joint tissue repair and regeneration [45].

Future Directions and Research Needs

Standardisation of protocols: Standardising protocols in preparing PRP and BMAC is imperative for enhancing the consistency and comparability of studies in regenerative medicine. Establishing uniform guidelines for the processing, concentration, and administration of PRP and BMAC ensures that researchers and clinicians adhere to a standard set of procedures. This not only streamlines data interpretation but also facilitates the identification of key factors influencing treatment outcomes. Standardised protocols contribute to the robustness of research findings, enabling a more accurate assessment of the efficacy and safety of PRP and BMAC in knee osteoarthritis [46].

Identification of biomarkers: The exploration of biomarkers holds significant promise for advancing regenerative therapies for knee osteoarthritis. Investigating specific biomarkers correlating with patient responsiveness to regenerative treatments allows for the development of personalised approaches. By identifying molecular or genetic indicators, clinicians can predict which patients will most benefit from PRP or BMAC interventions. This enhances treatment efficacy and aids in refining patient selection criteria, optimising resource utilisation, and minimising potential risks. Identifying biomarkers is critical for tailoring regenerative therapies to individual patient profiles, ultimately improving overall treatment outcomes [47].

Long-term follow-up studies: Long-term follow-up studies are essential for comprehensively evaluating the sustained efficacy and safety of regenerative therapies for knee osteoarthritis. While short-term outcomes provide valuable insights, understanding the durability of treatment effects and the occurrence of any latent adverse events is crucial for establishing the long-term viability of PRP and BMAC. Extended follow-up periods allow researchers to assess the persistence of symptomatic relief, functional improvement, and structural changes within the joint. These studies contribute valuable data to inform clinicians about regenerative therapies' durability and safety profile, guide clinical decision-making, and ensure the responsible integration of these treatments into long-term osteoarthritis management strategies [48].

Challenges and considerations in clinical practice

Standardization of Protocols

One of the critical challenges in the clinical application of PRP and BMAC for knee osteoarthritis lies in the standardisation of treatment protocols. The variability in preparation methods, concentrations, and delivery techniques can significantly impact therapy outcomes. Addressing this challenge involves establishing consensus guidelines for preparing and administering PRP and BMAC [49]. Standardisation efforts may encompass defining optimal platelet concentrations, white blood cell content, and activation procedures for PRP. Similarly, for BMAC, standardising the processing steps and ensuring consistency in the cellular composition are critical considerations. Achieving consensus on these protocols is essential to enhancing the reproducibility of results across different clinical settings, allowing for more reliable comparisons between studies and improved overall efficacy [50].

Patient Factors Influencing Treatment Outcomes

The success of PRP and BMAC therapies in knee osteoarthritis is intricately linked to patient-specific factors. Variability in patient demographics, disease severity, and underlying health conditions can influence treatment outcomes. Understanding and identifying these factors is imperative for tailoring regenerative medicine interventions to individual patient needs [51]. Factors such as age, comorbidities, and the stage of osteoarthritis may impact the response to PRP or BMAC treatment. Additionally, considering the presence of inflammatory conditions or systemic diseases is crucial for predicting and managing potential complications. Addressing patient-specific variables requires a personalised approach to treatment, emphasising the importance of thorough pre-treatment assessments and risk stratification [52].

Regulatory and Ethical Considerations

As regenerative medicine therapies gain popularity, navigating the regulatory landscape and adhering to ethical principles become paramount. Ensuring compliance with regulatory requirements is essential for maintaining patient safety and promoting responsible clinical practice. Regulatory considerations include approval processes for PRP and BMAC and adherence to guidelines for good clinical practice [53]. Ethical considerations extend to informed consent, transparency in reporting outcomes, and equitable access to these emerging therapies. Clinicians and researchers must navigate the ethical dimensions of offering PRP and BMAC, considering the balance between innovation and patient welfare. Exploring these regulatory and ethical considerations is crucial for establishing a framework that promotes the responsible and ethical integration of regenerative therapies into clinical practice [53].

## Conclusions

The comprehensive examination of PRP and BMAC in the context of knee osteoarthritis reveals significant findings that hold implications for clinical practice and direct recommendations for future research. PRP and BMAC exhibit promise as regenerative therapies, leveraging autologous components to address symptoms and potentially modify the disease trajectory. The nuanced understanding of their mechanisms of action, clinical efficacy, and safety profiles provides clinicians with valuable insights for informed decision-making in patient care. The implications for clinical practice underscore the need for a personalised approach, considering factors such as disease severity and individual patient characteristics. As these therapies integrate into the evolving landscape of osteoarthritis management, collaboration between healthcare providers becomes crucial. Future research should focus on longitudinal studies for assessing long-term outcomes, direct comparisons between PRP and BMAC, standardisation of protocols, identification of biomarkers, and cost-effectiveness evaluations. Addressing these research priorities will contribute to refining treatment strategies, enhancing patient outcomes, and establishing a more evidence-based approach to the regenerative management of knee osteoarthritis.
